# Induced Pluripotent Stem Cells; New Tools for Investigating Molecular Mechanisms in Anorexia Nervosa

**DOI:** 10.3389/fnut.2019.00118

**Published:** 2019-08-13

**Authors:** Gilles Maussion, Iveta Demirova, Philip Gorwood, Nicolas Ramoz

**Affiliations:** ^1^Montreal Neurological Institute and Hospital, McGill University, Montreal, QC, Canada; ^2^INSERM U1266, Institute of Psychiatry and Neuroscience of Paris, Paris, France; ^3^Hôpital Sainte-Anne (CMME), University Paris-Descartes, Paris, France

**Keywords:** anorexia nervosa, eating disorders, animal models, induced pluripotent stem cells, genetics, epigenetics

## Abstract

Anorexia nervosa (AN) is a dramatic psychiatric disorder characterized by dysregulations in food intake and reward processing, involving molecular and cellular changes in several peripheral cell types and central neuronal networks. Genomic and epigenomic analyses have allowed the identification of multiple genetic and epigenetic modifications highlighting the complex pathophysiology of AN. Behavioral and genetic rodent models have been used to recapitulate and investigate, with some limitations, the cellular and molecular changes that potentially underlie eating disorders. In the last 5 years, the use of induced pluripotent stem cells (IPSCs), combined with CRISPR–Cas9 technology, has led to the generation of specific neuronal cell subtypes engineered from human somatic samples, representing a powerful tool to complement observations made in human samples and data collected from animal models. Systems biology using IPSCs has indeed proved to be a valuable approach for the study of metabolic disorders, in addition to neurodevelopmental and psychiatric disorders. The manuscript, while reviewing the main findings related to the genetic, epigenetic, and cellular bases of AN, will present how new studies published, or to be performed, in the field of IPSC-derived cells should improve our current understanding of the pathophysiology of AN and provide potential therapeutic strategies addressing specific endophenotypes.

## Introduction: Anorexia Nervosa, a Multifactorial Disorder

Anorexia nervosa (AN), the psychiatric disorder with the highest suicide rate, estimated at 10% per decade, is prevalent in 0.5% of the general population and has a sex ratio of nine females to one male (1). While its genetic heritability has been estimated to be between 50 and 70% (2), environmental factors and epigenetic mechanisms are also thought to be involved in the physiology of AN. Investigations have been performed to analyze the genetic component of AN, either by gene candidate approaches (3, 4) or by genome-wide association studies (5, 6). However, replications of these studies did not necessarily validate previously discovered associations. In fact, the lack of consistency between replication studies has conducted physicians to reconsider the basis of AN, as well as the methods of diagnosis. In fact, whereas the diagnosis is mainly based on patient observation and food intake, most recent studies have demonstrated that AN would be more associated with deregulation of the reward system (7, 8). Genes that have been discovered to be involved in the pathophysiology of AN so far are related to neuroendocrine regulation, control of digestion, sleep, reward response, and neurotransmission. As the field of epigenetics has attracted a growing interest from the scientific community, especially in psychiatry, where most diseases are not completely determined by genetic causes, several studies have investigated methylation patterns in promoter regions of candidate genes in AN, such as *ANP* (9), *DAT1, DRD2* (10), *OXTR* (11), *POMC* (12), and *SNCA* (13), to better understand changes in the dopaminergic systems and hypothalamic pituitary adrenal (HPA) axis. One study investigated the genome-wide methylation profile of peripheral blood samples from individuals diagnosed with AN and evidenced differentially methylated regions in genes involved in development and brain plasticity including the dopaminergic and glutamatergic neurotransmission, and in RNA modifications (14). Although the molecular and cellular mechanisms involved in the pathophysiology of AN require further investigation, genetic and epigenetic studies have highlighted potential genes and mechanisms implicated in the psychiatric and metabolic pathways involved in AN. These developments have in turn promoted the increasing popularity of genetically engineered cellular and animal models for the study of eating disorders. The current review aims to present the added values of these models; and highlight new potential avenues that could be open by the use of human induced pluripotent stem cells (IPSCs).

## Imaging-Based Studies in Cohorts of Patients With Anorexia Nervosa

Brain imaging techniques such as functional magnetic resonance imaging (fMRI) and positron emission tomography (PET) have been used to better understand the interactions and connectivity between brain regions that may underlie impairments in reward processing, regulation in food intake, and executive functions describing AN (15). Dopaminergic and serotoninergic pathways respectively, involved (i) in reward processing and (ii) in the regulation of aggressive and impulsive behaviors, anxiety, alteration of body perception, and inhibition have been investigated to better decipher the interplay between food restriction and other behavioral traits observed in AN.

An fMRI study showed a group-specific activation of the ventral striatum in AN patients and controls submitted to visual stimuli related to body perception. A higher activation of the ventral striatum was observed in AN patients who received underweight stimuli (16). Interestingly, an increased skin conductance response (SCR) was observed in patients with AN submitted to underweight stimuli, suggesting a higher reward value of starvation in AN. Furthermore, within the AN group, the difference was more pronounced in the Met carriers of the brain-derived neurotrophic factor (BDNF) Val66Met polymorphism (4). The potential implication of genetic vulnerability factors related to the dopaminergic network and impairments in executive function such as inflexibility observed in AN was investigated in patients genotyped for the COMT Val158Met polymorphism, submitted to the Wisconsin Card Sorting Task and to a resting state fMRI. Patients with AN showed a higher level of perseveration, but only underweight patients showed cognitive dysfunction related to the VAL/MET polymorphism. Furthermore, MET/MET homozygous patients within the underweight group showed a higher activation in BA32. These data suggest that starvation affects dopamine degradation, which leads to cognitive impairments in the context of AN (17).

In another study, differences in connectivity were observed in the ventral attention network in patients with AN submitted to a stop–start paradigm, which evaluates motor response inhibition. The responses to external stimuli and connectivity are differentially correlated between controls and patients depending on the 5HTTLPR genotypes. This study suggests that the serotonin pathway may be involved in modulating response to environmental stimuli through changes in the ventral attention network activity in the context of AN (18).

In addition to fMRI-based analysis, studies using PET were performed to further characterize the activation of specific receptors and transporters. D2/D3 receptor binding with [(11)C]raclopride has been quantified in the brains of women who had recovered from AN using PET, with higher binding observed in the ventral striatum and which was correlated with harm avoidance (19). Other studies using PET have shown alterations in 5HT1A, 5HT2A, and 5HTT binding in the frontal, cingulate temporal, and parietal cortices, in the context of AN, which are maintained after recovery (20). These studies show that changes in serotoninergic and dopaminergic neurotransmission observed in AN persist after recovery.

The functional connectivity of brain regions was investigated by fMRI in patients with AN and bulimia who were submitted to a sucrose testing task. In AN, a loss of connectivity from the hypothalamus to the ventral striatum was observed, whereas in control conditions, these connections mediate reward processing related to food intake in response to appetite. In contrast, an increased connectivity between the orbitofrontal cortex and the ventral anterior insula was observed in AN and bulimia. These data suggest that food restriction observed in AN is related to a change in brain region connectivity. The reward related to food intake is supplanted by a decision-based food restriction (21).

## Animal Models of Eating Disorders and Their Limitations

Besides the human samples used to investigate the potential genetic and epigenetic components of eating disorders such as AN, animal models have been used to better understand the genes involved in and the mechanism that control the regulation of food intake, reward processing, and eating behaviors that may depend on environmental stress conditions.

The balance between satiety and appetite is in part controlled by neuro-hormones such as neuropeptide Y (NPY), leptin, and ghrelin, which are secreted by peripheral tissues and target brain regions such as the hypothalamus to control food intake. These neuro-hormones also contribute to the modulation of the stress response through changes in cortisol and insulin levels, both of which modify the dopaminergic response in the mesolimbic system, which is involved in reward processing (22). In animal models, it is mainly the levels of these neuro-hormones and hormones, and their effects on neuronal activity, that have been studied.

Deregulation of the reward system, accompanied by reduced food intake and increased activity, define AN and are largely captured in the activity-based anorexia (ABA) rodent model. This model, achieved by limiting access to food and offering unrestricted access to running wheels, results in weight loss and has revealed deregulations (i) in the opioid and dopaminergic reward circuitry, (ii) in the expression levels of hormones and neuro-hormones, and (iii) in the HPA axis, supporting observations in patients with AN (23). Rodents that have been submitted to ABA present increased endogenous levels of opioids concomitant with decreased food intake (24). Several studies have further investigated the dopaminergic activation in the ABA rodent model. Activation of the mesolimbic reward circuit in ABA rats rescues food intake without changing locomotor activity (25). Treatment of ABA mice with D2/D3 receptor antagonist was shown to decrease symptoms such as hypophagia and weight loss (26). Whereas BDNF, involved in neuronal development and synaptic plasticity, was shown to positively regulate D3 receptor expression in the striatum (27), its expression level assessed in the mesocorticolimbic reward circuit of ABA mice is specifically increased in the ventral tegmental area (28). Interestingly, *BDNF* heterozygous knockout (KO) mice present hyperphagia and obesity (29). These data suggest that control of food intake is interrelated with dopamine activation in the mesolimbic reward circuit. ABA mice also exhibit decreased levels of circulating leptin (positive regulator of food intake) and increased levels of ghrelin (orexigenic molecule), combined with an increased sensitivity to insulin (30). Interestingly, weight loss associated with ABA is reduced by ghrelin administration (31). Furthermore, a decrease of proopiomelanocortin (POMC) has also been observed in rats after 7 days under the ABA condition (32, 33), whereas plasmatic levels of corticosterone were decreased (34). In sum, the ABA model constitutes a stress condition, which leads to an increased secretion of glucocorticoids that modulate dopaminergic transmission in the reward circuit (35).

Models have also been developed to investigate the role of the stress component in eating disorders. Stress has been shown to affect the control of food intake through deregulations of the HPA axis (36). Decreased levels of peripheral ghrelin have been observed following activation of corticotropin releasing factor (37) in mice submitted to a novelty stress. Interestingly, direct administration of corticosterone in the nucleus accumbens, which leads to an increase of dopamine transient (38), is known to activate glucocorticoid receptors (GRs) in dopaminergic neurons from the mesolimbic region (39) and increase the appetite for psychostimulant drugs (40, 41). A study investigating an interaction between genes and the environment was performed on a mouse carrying the human BDNF Val66Met variant. Mice carrying the Met variant and submitted to social isolation at 7 weeks of age are more susceptible to present anorexia-like behavior (42). These data suggest that stressors can both affect the control of food intake and modulate the reward circuit. Another kind of stressor, maternal separation, if combined with a repeated fasting/refeeding cycle, has been shown to generate binge-like eating behaviors in adolescent rats (43). These findings indicate that the developed eating disorder may depend on the applied stressors.

Neuroendocrine changes that may occur in eating disorders have also been investigated in diet or in food restriction models. While hypoinsulinemia combined with an increased sensitivity to dopamine released in the striatum has been observed in animals submitted to chronic food restriction, increased food intake leads to the opposite phenotype (44). Food deprivation also affects the HPA axis by leading to increased levels of adrenocorticotropic hormone and a less efficient negative regulation mediated by corticosterone (45, 46).

Genetic models have also been generated to further understand gene deregulations in the context of eating disorders. The anorexic *anx/anx* mice, which carry a homozygous mutation on chromosome 2 (47), present as normal at birth but rapidly develop hyperactivity and a reduced food intake (48). Importantly, *anx/anx* mice under-eat despite having full access to food, in contrast to other models like ABA rodents. In these mice, peripheral levels of leptin are reduced, and the balance between anorexigenic and orexigenic peptides is disturbed (49–51), with larger populations of cells expressing NPY and agouti gene–related protein (AGRP) in the arcuate nucleus and lower levels of these in the hypothalamic and extra-hypothalamic target areas of the same neurons (52, 53). In humans, variants of AGRP are observed in patients with AN (54), and changes in NPY levels in cerebrospinal fluid are secondary to AN (55). The mice also develop inflammation and degeneration in brain areas near neurons expressing AGRP (56, 57) and have dysfunctional pancreases, resulting in glucose intolerance and high levels of circulating free fatty acids (58)—mirroring findings in people with AN (59). However, one limitation of *anx/anx* mice is that they die approximately 1 month after birth due to mitochondrial dysfunction and neurodegeneration (47).

Similarly, mice with a conditional KO for the tyrosine hydroxylase (*Th*) gene in dopaminergic neurons (60) died 1 month after birth from starvation. These mice were also hypophagic and hypoactive. The lack of food intake in mice with impaired dopamine production suggests interplay between feeding behavior and dopamine-mediated reward processing. While animal models have already provided a substantial amount of information on the mechanisms that regulate food intake and reward processing, it remains very difficult to determine how these two functions, seemingly intersecting each other in the context of eating disorders, are connected in humans. Another limitation remains in matching biological and behavioral observations in these models with the clinical criteria used for diagnosing people with such disorders. Finally, eating disorders such as AN are recognized to be multifactorial diseases, arising from a variety of genetic and epigenetic factors that cannot be fully recapitulated in animal models, where the roles of different genetic and environmental factors can only be studied in relative isolation.

Data collected from genetic, epigenetic, and imaging-based studies on cohorts of patients with AN, and on rodent models, provide information and potential mechanistic insights that can be further investigated and complemented by research performed on IPSCs.

## IPSC-Derived Cells and Eating Disorders

Whereas, most of the studies performed in the field of AN have primarily used DNA to analyze either potential genetic vulnerability or methylation profiles, somatic cells can also be used to further investigate changes observed in any cell type of interest. In fact, somatic cells such as fibroblasts or peripheral blood monocyte cells can be reprogrammed into IPSCs by overexpressing four transcription factors, *KLF4, C-MYC, OCT4*, and *SOX2* (61). The IPSCs can be induced to neurons or other cell types of the central nervous system (CNS) (62) or other organs. IPSC-derived cells have notably been used to investigate neurodevelopmental disorders (63, 64) for which post-mortem brain tissue is not necessarily accessible.

Hypocretin (HCRT) or orexin is a neuropeptide expressed in the hypothalamus that positively regulates food intake (65). A recent study has been able to generate orexin positive neurons derived from human IPSCs. The authors demonstrated that, in the IPSC-derived neurons, prolonged exposure to glucose induces a silencing of the *HCRT* gene mediated by histone modifications, specifically H3/H4 hypoacetylation. Interestingly, cell treatment with N-acetyl-d-mannosamine, a derivative metabolite produced from glucose, reactivates *HCRT* expression in IPSC-derived neurons (66).

IPSCs have recently been used for the first time to investigate changes in gene expression profiles that may occur in the context of AN (67). The authors performed an RNA sequencing analysis on 4-week differentiated IPSC-derived neurons from controls and AN patients. The IPSC-derived cells obtained were mostly glutamatergic neurons. The authors found that, after correction for multiple testing, in a list of 110 differentially expressed genes (DEGs), some of them encode proteins that are likely to interact together. A functional annotation of the DEG showed an enrichment of genes involved in the tachykinin receptor pathway and in the response to estrogen. As the tachykinin receptor is expressed in dopaminergic structures such as the striatum (68), the change in expression of *TACR1* is likely related to an alteration in reward processing, which is dysregulated in AN. Furthermore, several studies have demonstrated that the tachykinin receptor is likely to mediate a reward response to opioids (69) and alcohol intake (70, 71), and its mediated pathway has being involved in the addiction process (72). Data collected by Negraes et al. provide supplementary evidence that neuroendocrine responses and dopaminergic neurotransmission related to reward processing are altered in AN. Whereas, Negraes et al. studied IPSC-derived glutamatergic neurons from patients, results collected from brain imaging studies on patients with AN and from rodent models have shown changes in serotoninergic and dopaminergic pathways and neurons in the context of AN. These two neuronal cell subtypes, derived from human IPSCs, have to be further investigated in the context of AN (beginning with their transcriptome and methylome), as procedures to generate them are now available (73, 74). Many drugs targeting serotoninergic and dopaminergic neurotransmission have been used to improve behavioral features related to AN (75). Such drugs should be tested on IPSC-derived cells from patients (i) to determine the functional effect of the drug on a cellular and molecular level and (ii) to infer the patient response. On the functional side, as the ghrelin receptor has been shown to form a heterodimer with dopaminergic receptors to mediate the anorexigenic effect of ghrelin (76), the effect of the neuro-hormones on (i) neuronal activity of the dopaminergic network and (ii) downstream signaling pathways could be investigated using IPSC-derived dopaminergic cells.

Patient IPSCs make it possible to generate and analyze specific cell subtypes that contain most or all of the genetic and epigenetic information potentially relevant to AN, whereas animal models cannot capture the multigenic risk factors mediated by the patient's genetic background.

## Investigating Epigenetic Regulation in IPSC-Derived and Induced Neuronal Cells

As epigenetic regulation seems to play a crucial role in AN, being able to generate IPSC-derived neuronal cells from patient samples would permit the analysis of cell type–specific differential methylation related to this eating disorder. A database was recently created containing the complete DNA methylation profiles of blood and brain samples of 16 control individuals (77). Such databases could be used to compare methylation profiles between patient samples and IPSC-derived cells. Regarding the analyses of DNA methylation in IPSC-derived cells, several studies investigating genetically defined neurodevelopmental disorders have highlighted relevant differentially methylated regions that can be compared with genome-wide expression profiles (78–80) and potential disease phenotypes. However, it is important to note that during the reprogramming of somatic cells to IPSCs, the original methylation pattern can be partially lost in specific DNA regions (81–83). Although IPSC-derived cells represent a very powerful tool for the investigation of genetically defined eating disorders, some limitations may be encountered for eating disorders whose genetic component has not been well-established. In order to minimize changes in the methylation patterns during cellular reprogramming, procedures to directly convert blood cells to neurons are currently being improved (84). Such procedures constitute a new strategy to investigate *in vitro* neuronal models of large cohorts. Furthermore, changes in global DNA methylation profiles have been analyzed during the direct reprogramming of fibroblasts to neurons (85) in order to identify modifications of methylation patterns that are specific to those procedures. Relevantly, several studies have highlighted changes in DNA methylation profiles from AN patients' blood cells (86). While the CRISPR–Cas9 system has primarily been used in IPSCs to edit the genome and create or repair mutations within an isogenic background, the system, when coupled to the DMNT3A or TET1 enzymes, is capable of methylating or demethylating specific sequences. The procedure, initially tested in transformed cells lines (87, 88), has been adapted to IPSCs (89, 90). As an example, targeted methylation of Exon 1 of the *SNCA* gene [whose promoter was found to be hypermethylated in AN (13)] on IPSC-derived dopaminergic neurons led to decreased expression of synuclein alpha (SNCA) (91). IPSC-derived and induced neuronal cells from patients, combined with CRISPR systems, could allow for (i) the identification of specific epigenetic signatures related to dysregulations and behaviors observed in AN, (ii) the investigation of specific treatment responses, as well as (iii) the observation of cellular and molecular phenotypes after restoration of the DNA methylation profile.

## Conclusion

Eating disorders, including AN, are complex and multifactorial diseases. Investigations performed on cohorts of patients suffering from eating disorders have shown potential genetic associations and epigenetic changes related to the control of food intake and reward processing. Furthermore, animal models have played a key role in beginning to decipher the role of hormones and neuro-hormones in these two affected mechanisms. However, these approaches do not sufficiently permit the investigation of the interplay between regulation of food intake and reward processing and do not recapitulate the complex behavioral phenotypes observed in patients with AN, including life events and the chronicity of this disorder. With this in mind, while methylation changes during the reprogramming steps need to be carefully examined, patient IPSC-derived cells, such as serotoninergic and dopaminergic neurons, combined with CRISPR editing tools, constitute potentially powerful models to further analyze the cellular and molecular mechanisms, gene expression changes, and epigenetic modifications that underlie eating disorders.

## Author Contributions

GM, NR, and PG designed the manuscript. GM, NR, and ID wrote the manuscript. ID, NR, and PG edited the manuscript.

### Conflict of Interest Statement

PG received during the last 5 years fees for presentations at congresses or participation in scientific boards from Alcediag-Alcen, AstraZeneca, Bristol-Myers Squibb, GSK, Janssen, Lilly, Lundbeck, Otsuka, and Servier, none of these being related to the present study. The remaining authors declare that the research was conducted in the absence of any commercial or financial relationships that could be construed as a potential conflict of interest.
